# Medical Items

**Published:** 1914-04

**Authors:** 


### MEDICAL ITEMS

### BUDWELL'S—THE PALATABLE EMULSION.

One of the main factors in giving Budwell's Emulsion of Cod Liver Oil its reputation for palatability is that the Emulsion is prepared from the sweetest and freshest Norwegian cod liver oil, and the shipments are so arranged that orders are always filled from new fresh stock.

The delicate stomachs of even young children have no trouble with Budwell's Emulsion and yet all the alterative and nutritive properties of the oil are retained, with the disagreeable features climinated.

Budwell's Emulsion No. 1 (plain with the iodides) and Emulsion No. 2 (identical with the No. 1 with creosote carbonate and purified guaiacol added) has been offered the profession for twenty years now by the manufacturers, The Budwell Pharmacal Co., of Lynchburg Virginia, for their use where a remedial agent in respiratory and pulmonary troubles is indicated and where a remedy is desired in all debilitated states. This company only appeals and advertises to the medical profession, to whom they will send samples; and copies of endorsements from the leading medical men of this country.

